# Escalated begging does not compromise nestling health

**DOI:** 10.1093/beheco/araf003

**Published:** 2025-01-28

**Authors:** Daniel Parejo-Pulido, Tomás Redondo, Silvia Casquero, Lorenzo Pérez-Rodríguez

**Affiliations:** Instituto de Investigación en Recursos Cinegéticos (IREC), CSIC-UCLM-JCCM, Ronda de Toledo 12, 13005 Ciudad Real, Spain; Estación Biológica de Doñana (EBD), CSIC, Americo Vespucio 26, 41092 Sevilla, Spain; Instituto de Investigación en Recursos Cinegéticos (IREC), CSIC-UCLM-JCCM, Ronda de Toledo 12, 13005 Ciudad Real, Spain; Instituto de Investigación en Recursos Cinegéticos (IREC), CSIC-UCLM-JCCM, Ronda de Toledo 12, 13005 Ciudad Real, Spain

**Keywords:** communication, costly signaling, growth, immunity, oxidative stress, parent-offspring conflict

## Abstract

A widely accepted explanation for the reliability of offspring begging signals assumes a differential benefit model balanced by direct viability costs independent of offspring nutritional condition. However, supporting evidence for this idea is inconclusive and often hampered by methodological limitations, including differential stimulation protocols and reliance on single, potentially biased markers of nestling health. This study tested the existence of direct, intrinsic, and condition-independent allocation trade-offs between begging and body mass, immunity and oxidative stress by manipulating the begging effort of spotless starling (*Sturnus unicolor*) nestlings while maintaining constant food intake. We addressed potential problems of previous experimental protocols, ensuring uniform stimulation levels and evaluating multiple immune and oxidative markers. We observed no significant effects of experimentally increased begging effort in any of the 14 physiological markers analyzed, with 95% confidence intervals of effect sizes consistently including zero or one (for the lysis capacity of plasma), indicating no biologically relevant effects. Overall, our findings suggest no physiological trade-offs associated with intense begging.

## Teaser

This study challenges a widely accepted model of begging signals, suggesting honesty is maintained because escalated begging compromises other vital processes. By experimentally increasing begging effort in spotless starling nestlings and assessing multiple health indicators, we found no evidence that begging affects mass gain, immune function or oxidative status. These findings call for a re-evaluation of traditional theories on honest begging signals.

## Introduction

The problem of reliability presents a significant theoretical challenge for understanding the evolution of animal communicative signals (Maynard-Smith and Harper 2004). The costly signaling hypothesis posits that animal signals evolve as reliable indicators of quality because their production or maintenance incurs risks or diverts resources from other essential processes in a way that only high-quality individuals can afford (Grafen 1990; Biernaskie et al. 2018). Begging signals, which honestly reflect offspring food requirements and regulate parental feeding responses in the context of parent-offspring conflict, have become a recurrent and suitable model system for studying animal communication (Wright and Leonard 2002). Godfray’s model of differential benefits (Godfray 1991, 1995; Johnstone 1997), inspired largely by avian begging, is the most influential model of costly signaling in parent-offspring communication (Mock et al. 2011). According to this model, at the honest equilibrium, offspring signal intensity is strictly determined by their nutritional condition. Offspring in poorer condition invest in greater signaling because they accrue higher fitness benefits from obtaining extra food through more intense begging displays. Here, signal cost increases monotonically with higher signal intensity, regardless of the offspring’s condition. This contrasts with another family of costly signaling models, where signalers accrue similar benefits but differ in the marginal cost of signaling (differential cost models) (Grafen 1990). The concept of fitness costs presents both theoretical and empirical challenges (Moreno-Rueda 2007; Számadó 2011; Higham 2014). Traditional signaling models proposed an intrinsic cost at equilibrium, either condition-dependent (Grafen 1990) or independent (Godfray 1991). However, recent “trade-off” models (Számadó et al. 2022, 2023) show that no equilibrium cost is required. Honesty is maintained if the marginal cost of out-of-equilibrium signals exceeds their marginal benefits, allowing for cost-free or even beneficial signals at equilibrium (Számadó et al. 2022, 2023). Although both models rely on trade-offs, traditional models were misinterpreted as “handicap models,” leading to confusion (Penn and Számadó 2020).

In Godfray’s model, three different trade-offs moderate signal overplay (Godfray 1991, 1995). First, a trade-off exists between direct and indirect components of nestling inclusive fitness, where escalated begging may reduce the number or quality of present or future siblings (Johnstone and Grafen 1992; Nöldeke and Samuelson 1999). Second, a direct resource allocation trade-off (Van Noordwijk and De Jong 1986) occurs between components of viability if intense begging, involving vigorous posturing and calling, diverts resources from growth, immune function, or other vital processes (Kilner 2001; Moreno-Rueda 2010). Finally, a third trade-off between viability and predation risk has been proposed: loud begging or intense parental activity may attract predators to the nest (Martin et al. 2000; Magrath et al. 2010), an extrinsic direct cost (Godfray 1995). Although the role of extrinsic direct costs—which are shared by all siblings regardless of their individual begging levels—in model stability is unclear (Godfray 1995), intrinsic direct costs remain essential in Godfray’s model.

Over the past four decades, significant effort has been made to find empirical evidence of intrinsic trade-offs in begging as predicted by costly signaling theory. Early studies concluded that the metabolic expenditure of avian begging, measured by oxygen consumption, is low (Chappell and Bachman 2002). However, these measurements ignored anaerobic metabolism (Weathers et al. 1997) and are not easily translated into fitness (Royle et al. 2004). More recent studies attempted to quantify begging costs in terms of growth rates, a common proxy for nestling fitness (Perrins and McCleery 2001). The results were mixed and inconclusive: some studies found negative effects of begging on offspring growth (Kilner 2001; Rodríguez-Gironés et al. 2001; Moreno-Rueda and Redondo 2011; Moreno-Rueda et al. 2012; Soler et al. 2014; Nettle et al. 2017), while others did not (Kedar et al. 2000; Rodríguez-Gironés et al. 2001; Leonard et al. 2003; Moreno-Rueda and Redondo 2012; Redondo et al. 2016), even when testing the same species.

A second wave of studies examined other physiological trade-offs related to nestling viability that proved relevant to costly signaling theory in the context of sexual signals as honest indicators of male health (Roberts et al. 2004; Hill 2011). Notably, begging consistently impacted immune function across five bird species. Studies have shown that intense begging reduces nestling cell-mediated immune response to a single antigen (Moreno-Rueda 2010; Moreno-Rueda and Redondo 2011, 2012; Moreno-Rueda et al. 2012; Redondo et al. 2016) and affects two inflammatory markers in adulthood, particularly in interaction with food conditions during the nestling stage (Nettle et al. 2017). However, these studies have two major limitations. First, they employed protocols where experimental nestlings were forced to beg longer than controls by receiving more stimulation from researchers, potentially causing both higher begging effort and observer-induced corticosterone-mediated stress responses, which could lead to immunosuppression (Saino et al. 2003). Second, these studies focused on only one or two markers of immune function, which is insufficient to capture the immune system’s complexity (Norris and Evans 2000; Keil et al. 2001; Adamo 2004).

In addition, begging activity can increase the production of reactive oxygen species, altering oxidative status and leading to oxidative damage (Noguera et al. 2010). Nestlings that beg intensively may reduce other pro-oxidant life-history components, such as growth and immune response, in order to maintain their oxidative balance (Alonso-Álvarez et al. 2007; Sorci and Faivre 2008; Costantini and Møller 2009). Moreno-Rueda et al. (2012) identified a three-way trade-off where prolonged begging increased oxidative damage, detectable only after controlling for mass gained and intensity of immune response. Nettle et al. (2017) found that begging positively affected oxidative DNA damage in adulthood and accelerated telomere attrition, a marker of aging related to oxidative stress (Armstrong and Boonekamp 2023). Other experimental studies manipulating nestling oxidative status (rather than begging effort) and then measuring begging performance showed mixed results, with some reporting positive effects (Noguera et al. 2010; Fuertes-Recuero et al. 2024), context- and sex-dependent effects (Boncoraglio et al. 2012; Maronde et al. 2018; Parejo-Pulido et al. 2023), or no effects at all (Hall et al. 2010; Maronde and Richner 2015; Fuertes-Recuero et al. 2024). In addition, other mixed results found by correlational studies (Boncoraglio et al. 2012) should be taken cautiously, as multiple simultaneous trade-offs may not all be measured (Lachmann et al. 2001; Moreno-Rueda 2007; Számadó 2011).

Despite testing various physiological trade-offs in numerous species, empirical evidence of avian begging costs remains inconclusive (Számadó et al. 2019). This study aims to test for direct, intrinsic, and condition-independent trade-offs between begging and various measures of nestling health related to viability. As predicted by Godfray’s model, we expect these trade-offs to be detectable with increasing signal intensity while keeping condition and benefits constant (Godfray 1991, 1995). We manipulated begging effort by forcing experimental nestlings to beg longer than control nestmates while maintaining equal food amounts for similar-sized nestlings on an identical schedule. As viability proxies, we evaluated growth, immune function, oxidative status, and corticosterone levels. Our protocol addresses limitations of previous studies by (1) ensuring similar stimulation levels for control and experimental nestlings to avoid differential glucocorticoid-mediated stress responses, and (2) simultaneously assessing multiple immune and oxidative status biomarkers.

## Materials and Methods

### Experimental design

The study was conducted during the spring of 2022 in a nest-box breeding population of spotless starlings (*Sturnus unicolor*) in central Spain (Soto del Real, Madrid). Spotless starlings are medium-sized colonial passerines that typically lay two asynchronous clutches per season, with a modal brood size of four nestlings (Veiga and Polo 2016). Hatching dates were determined through regular inspections. Procedures were overseen by the Animal Welfare Body of the University of Castilla-La Mancha and approved by the competent authority of the Junta de Comunidades de Castilla-La Mancha (approval reference: PR-2020-03-08), thus complying with current European, Spanish and Institutional laws on the care and use of animals.

The experiment involved 58 nestlings from 29 nests at the end of the linear growth phase (9 to 10 d after hatching, Muriel et al. 2021). In the afternoon preceding the experiment (at 8 d of age), we selected two chicks of intermediate mass rank from each brood, ensuring at least two nestlings remained to prevent parental desertion. Initial blood samples (350 µl) were taken from the jugular vein of the focal chicks to measure basal levels of immune and oxidative markers. We also weighed the nestlings (accuracy = 0.1 g) and randomly assigned them to either a high-begging (HB) or low-begging (LB) treatment. Nestlings were then transported in a warm container to the laboratory, where they were placed in individual nest cups inside an incubator (RCOM 50 PRO, Autoelex, Korea) with controlled temperature (27 to 33 °C) and humidity (55% to 60%), ensuring identical environmental conditions and potential stressors. That afternoon, nestlings were conditioned to a standard begging stimulus (a playback of a parental nest-feeding call recorded from the same population) while being fed crickets (*Acheta domestica*) ad libitum.

Early the next morning (day 9), nestlings were weighed after the first meal of the day. We estimated the daily food intake based on their mass, following the allometric relationship by Weathers (1996): daily food intake = 0.98  M^0.814^, where M is nestling body mass in grams. The daily food was divided into 12 equal portions, corresponding to 12 begging sessions conducted every hour from 8:00 to 20:00. Feeding intervals were based on mean intervals of parental feeding rates per nestling obtained from continuous recordings of 48 nests aged 6 to 8 d in our study population (mean interval ± SE = 59.05 ± 4.27 min). Any deviations from expected food intake were compensated for in subsequent sessions. The food consisted of alternating crickets and tiny omelette chunks (boiled egg), each weighed individually (Redondo et al. 2016). Whole egg is considered a high-quality diet (Moreno-Rueda and Redondo 2012), while insects are the bulk of the natural diet for nestlings (Veiga and Polo 2016).

During each feeding session, both nestlings in each dyad were carried together, covered by a cloth, to an adjacent room and stimulated to beg using the same playback of a feeding call. Each session included 1 to 4 trials in a random sequence determined beforehand as in Rodríguez-Gironés et al. (2001). Transporting both nestlings together ensured they experienced the same stressors and acoustic stimulation, which can influence glucocorticoid levels. To induce differences in begging effort, LB nestlings were fed immediately after gaping, while HB nestlings were stimulated to beg repeatedly by playing the playback and placing forceps over their mouths for 30 s longer than LB nestlings, resulting in a maximum cumulative duration of 2 min more per session in the 4-trial sessions. This duration was chosen to ensure differences in hourly begging rates between experimental groups comparable to previous studies assessing the physiological impact of begging. Our protocol resulted in differential begging rates higher than 45.5% of previous studies (Leonard et al. 2003; Moreno-Rueda and Redondo 2011, 2012; Moreno-Rueda et al. 2012; Redondo et al. 2016), exceeded by six studies (Soler et al. 1999; Kilner 2001; Rodríguez-Gironés et al. 2001; Moreno-Rueda 2010; Soler et al. 2014; Nettle et al. 2017).

Two 4-trial begging sessions (at 11:00 and 17:00 h) were recorded with a digital video camera (Panasonic HC-V180) to validate the effects of the experimental manipulation on begging effort. A trained observer, blind to the experiment’s purpose and chick treatments, analyzed the videos at a reduced speed (0.125x) using Solomon Coder software (Version: beta 19.08.02, Péter 2013). Postural begging intensity was assessed on a five-level ordinal scale: 0 (no begging), 1 (gaping, neck flexed, body horizontal), 2 (gaping, neck extended, body horizontal), 3 (gaping, neck extended, body off the nest floor, back reclining), and 4 (fully stretched, vertical back, sometimes wing flapping) (adapted from Redondo and Castro 1992). For each trial, we recorded the time spent by the nestling at each postural intensity level and calculated the total duration of the begging bout by summing these durations. A summed begging score was computed by multiplying the duration of each begging level by its rank and summing them (Leech and Leonard 1996; Kilner 2001). The mean postural intensity was obtained by dividing the summed begging score by the total duration. Nestlings were weighed again at the end of the day.

The same procedure was repeated on day 10, except that after the last begging session, a 350 µl blood sample was collected from the jugular vein to analyze post-treatment immune and oxidative stress markers. All samples from both days 8 and 10 were collected while recording the time elapsed between opening the nest box or the incubator and sample collection (always < 3 min) to minimize disturbance effects on corticosterone (CORT) values (Jimeno et al. 2017). Samples were stored at 4 °C until centrifugation (5 min at 4 °C and 9,000 g) within 2 h after collection. Plasma and pellet were separated, the pellet was washed with cold saline solution, and both fractions were frozen at –80 °C until analysis. Nestlings were returned to their original nests the following morning (day 11). At this moment, one randomly selected non-experimental nestmate was also blood-sampled within 3 min after opening the nest box to analyze potential differences in CORT levels due to laboratory procedures.

To verify that our experimental manipulation induced elevated begging in HB nestlings within the range of natural begging durations, we measured individual durations of begging bouts in 164 feeding events from 41 broods 9 to 10 d-old, recorded using an infrared video camera (BOBLOV PD50-32GB).

### Immunological and oxidative stress assays

We quantified several immunological markers involving cell-mediated, humoral, and integrated immunity of both innate and acquired components. Specifically, we assessed the inflammatory T-cell mediated immune response elicited by an antigen using the phytohemagglutinin (PHA) skin test (Salvante 2006); the cellular capacity to fight infections and other diseases by quantifying the number of white blood cells (WBC) relative to 10,000 erythrocytes and the ratio of heterophils to lymphocytes (H:L ratio) in blood smears (Salvante 2006); complement activity and natural antibody levels using the lysis/agglutination assay (Matson et al. 2005); the acute-phase response to infection, inflammation, or trauma by measuring plasma haptoglobin levels (Millet et al. 2007); and plasma capacity to defeat micro-organisms by quantifying bacterial (*Escherichia coli*) killing capacity (Matson et al. 2006).

We assessed oxidative stress by targeting different components of the system with several biomarkers of oxidative damage and antioxidant mechanisms (Halliwell and Gutteridge 2007; Monaghan et al. 2009; Pérez-Rodríguez 2009). Specifically, we measured a marker of oxidative damage by quantifying a byproduct of lipid peroxidation in plasma (malondialdehyde acid, MDA) (Mateos and Bravo 2007); two markers of the combined action of non-enzymatic antioxidants in plasma, the Trolox Equivalent Antioxidant Capacity (TEAC) (Miller et al. 1993) and the OXY-adsorbent Assay (Costantini 2011); the absolute levels of a key intracellular endogenous antioxidant (total glutathione, tGSH) in erythrocytes; and an oxidative status index based on the functional levels of that antioxidant, the reduced to oxidized glutathione ratio (GSH:GSSG ratio) (Halliwell and Gutteridge 2007).

We quantified plasma CORT levels to ensure our stimulation protocol caused similar stress levels between both nestling groups and to control for potential effects of the levels of this hormone on begging and physiological variables. Detailed protocols for these techniques are available in the Supplementary data.

In our study, we rigorously followed a blind methodology for the PHA skin test, as measurements were made by a single person (S.C.) entirely unaware of nestling identity and previous laboratory procedures. Additionally, biochemical analyses were conducted blind to the experimental treatments, with samples from each dyad run in the same session in random order.

### Statistical analyses

We performed statistical analyses using R 4.3.1. To enhance model stability, convergence likelihood, and accuracy of parameter estimates, we Z-transformed all continuous independent variables (mean-centered with an SD of 1) (Harrison et al. 2018).

To verify that our experimental manipulation (HB and LB treatment) created the expected differences among groups, we conducted separate linear mixed models for time begging, begging score, mean postural intensity, and time spent at high postural intensity levels (4 and 5) as response variables, with the experimental treatment as the main predictor. We included the order of the begging trial (up to 4), the begging session (11:00 or 17:00 h), and the day (day 9 or 10) as covariates, with nestling ID nested within the nest of origin and the date of arrival at the laboratory as random effects. We also assessed food consumption by nestlings using the total mass of food consumed (boiled egg and crickets) during the two-day experiment as the response variable. This model included the experimental treatment as the main predictor, initial body mass as a covariate, and nestling ID nested within the nest of origin and the date as random effects.

To assess the effect of the begging manipulation on body mass, corticosterone levels, immune function (PHA skin test, total WBC count, H:L ratio, lysis and agglutination capacity, haptoglobin levels, bacterial killing capacity), and oxidative stress markers (MDA, TEAC, OXY, tGSH, GSH:GSSG ratio), we used linear mixed models for each response variable. Lysis capacity was analyzed using a generalized mixed model with a binomial error distribution due to low variability (76 samples had titers of 0, 28 of 1, and only 2 samples had titers of 2).

To control for initial levels of each physiological marker, we included the experimental treatment (HB or LB) in interaction with the time of the measurement (pre- or post-treatment) as the main predictor to analyze the change due to the treatment (Repeated measurements model). Initial mass (day 8) was included in all models as a covariate (except when mass was the response variable). All models included nestling ID nested within the nest of origin and the date of arrival at the laboratory as random effects. For the PHA skin test model, treatment was the only predictor since only one measure was taken per nestling (day 10), and nestling ID was therefore removed as a random effect. Additional covariates included handling time (time from opening the nesting box or incubator until blood collection) for the CORT model and levels of uric acid for the TEAC model (Cohen et al. 2007). Differences in MDA levels according to treatment and time were also checked, considering triglyceride levels as covariate (Pérez-Rodríguez et al. 2015). Failures to obtain sufficient blood volume in some individuals led to varying sample sizes between markers. We removed the entire dyad from the analysis of that marker if any measurement was missing (either initial or final values).

To investigate potential time-dependent effects on mass gain (Soler et al. 2014; Redondo et al. 2016), we conducted separate models testing differences in body mass on the first (day 9) and last day of the experiment (day 10). In these models, the variable “time of measurement” was a categorical factor with two levels (beginning or end of the day).

We confirmed the robustness of our results by means of two other additional analytical approaches. First, we replaced the categorical factor “Treatment” with a continuous variable accounting for the average time each nestling spent begging. Second, we used nestling dyads as our experimental unit and modeled how final HB-LB differences in physiological variables were explained by differences in begging as the main predictor (Within-Dyad differences model) (Kilner 2001; Leonard et al. 2003). Both approaches account for individual variability in begging effort within each “Treatment” level and inconsistencies in creating uniform differences in begging effort between HB and LB across all dyads (see Supplementary data for details).

To address multiple testing when analyzing how experimental treatment affected multiple physiological variables, we applied a *P*-value correction using the Benjamini-Hochberg method to control for false discovery rate in begging, growth, immunological, and oxidative stress analyses (Benjamini and Hochberg 1995; Storey and Tibshirani 2003).

To measure the magnitude of the effects found in our study, we calculated effect sizes [adjusted standardized mean differences for dependent samples (Hedges’ g), and odds ratio (OR) for the probability of lysis capacity] with their 95% confidence intervals (CIs), following recommendations by Nakagawa and Cuthill (2007) and Lakens (2013), using the *esc* R package (Lüdecke 2019). Additionally, we report standardized beta coefficients (β) with 95% CIs from our models to consider the influence of other factors (initial values, covariates, random effects) on the effect of the treatment on the physiological variables. These coefficients estimate standardized effect sizes for fixed effects in multiple regressions (Schielzeth 2010). We considered effect sizes whose CIs included zero (for Hedges’ g or β) or one (for the odds ratio) as “no-effect” (Nakagawa and Cuthill 2007; Tenny and Hoffman 2017).

## Results

Prior to the experimental manipulation, LB and HB nestlings showed no significant differences in initial mass (day 8), CORT levels, or immunological and oxidative stress markers (all corrected *P* > 0.460; Table S1).

**Fig. 1.**
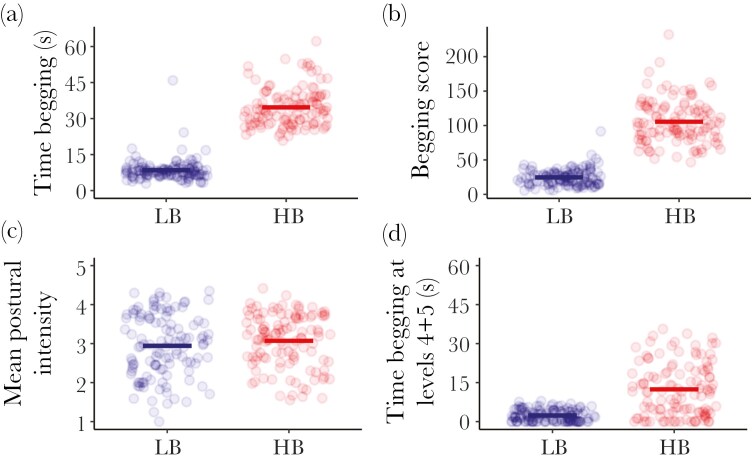
Effect of experimental treatment (LB or HB) on the a) time begging, b) begging score, c) mean postural intensity, and d) time begging at levels 4 and 5. Each point is the raw value for each individual in each begging trial. Bold horizontal lines are the means for each group.

Both LB and HB nestlings consumed the same total amount of food during the experiment (mean ± SE: HB: 48.8 ± 0.75 g; LB: 49.3 ± 0.75 g; F_1,28_ = 2.49, *P* = 0.126; Table S2). The experimental treatment successfully made HB nestlings beg much longer per trial than LB nestlings (F_1,28_ = 651, corrected *P* < 0.001; Fig. 1a; Table S2), with higher postural intensity scores (F_1,28_ = 760, corrected *P* < 0.001; Fig. 1b; Table S2), mean postural intensities (F_1,28_ = 95, corrected *P* < 0.001; Fig. 1c; Table S2), and time spent at high postural intensity levels (4 and 5) (F_1,28_ = 92, corrected *P* < 0.001; Fig. 1d; Table S2). HB and LB nestlings showed average begging durations of 97.0 ± 14.4 s/h and 26.5 ± 7.28 s/h, respectively. The mean duration of begging bouts per trial of LB nestlings (mean ± SE = 9.34 ± 0.48 s, Fig. 1) was slightly lower than the duration of begging bouts per parental visit in same-age wild broods from the same population (mean ± SE = 12.3 ± 0.68 s, range = 1.0 to 33.1 s; Wilcoxon rank-sum test: W = 1567, *P* = 0.011, Hedges’s g = 0.54 [0.05, 0.97]). HB nestlings had mean begging bouts (mean ± SE = 34.2 ± 0.94 s, Fig. 1) 3.7 times longer than LB nestlings and much longer than those from wild broods (Wilcoxon rank-sum test: W = 16, *P* < 0.001, Hedges’s g = –3.67 [–4.31, –3.02]), being slightly above the maximum bout duration recorded in the wild (33.1 s). Notably, the manipulation significantly increased hourly begging durations of HB nestlings (Wilcoxon rank-sum test: W = 366, *P* < 0.001; Hedges’s g = –1.09 [–1.54, –0.65]) to 1.7 times the mean hourly durations of wild broods (mean ± SE = 58.7 ± 39.4 s, range = 4.1 to 210 s), though still within the observed natural range. Hourly begging durations of LB nestlings were also lower than those from wild broods (Wilcoxon rank-sum test: W = 1961, *P* < 0.001, Hedges’s g = 0.94 [0.40, 1.38]).

### Effect of begging on body mass, CORT, immunological, and oxidative stress markers

**Fig 2.**
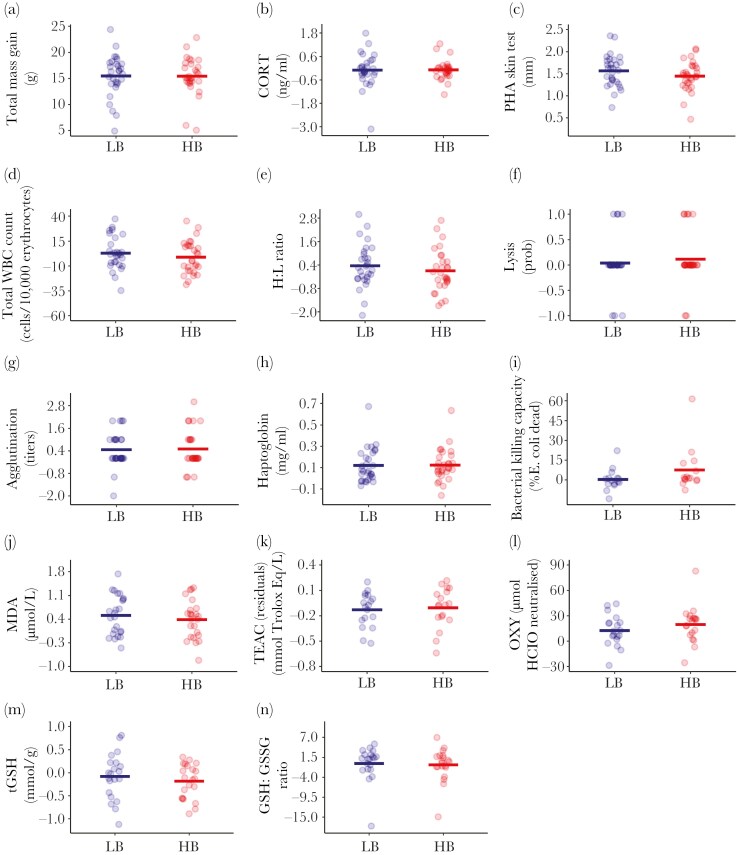
Effect of experimental treatment (LB or HB) on a) total mass gain, b) CORT, c) PHA skin test, d) total WBC count, e) H:L ratio, f) probability of lysis, g) agglutination, h) haptoglobin levels, i) bacterial killing capacity, j) MDA levels, k) TEAC, l) OXY, m) tGSH levels, and n) GSH:GSSG ratio between initial and final measurements. In panels A-B, D-N, each point is the difference between initial and final raw values for each individual, whereas in panel C points represent values of patagium swelling at the end of the experiment. Panel K represents the difference of residuals of TEAC after controlling by uric acid levels between the initial and final measurements. Bold horizontal lines are means for each group.

HB and LB nestlings did not differ in body mass gain during the experiment (Table 1 and Fig. 2a; Table S3). Analyzing body mass change as differences between HB and LB nestlings for each day also revealed no significant treatment effect (Table S3). LB and HB did not differ in basal CORT levels, indicating similar environmental stressors and stimulation levels due to the experimental manipulation (Table 1 and Fig. 2b; Table S3). Additionally, laboratory conditions did not increase basal CORT levels compared to unmanipulated siblings in natural nests (Table S4).

**Table 1.** Standardized beta (β) coefficients (with 95% CI), F tests (with df), and uncorrected and corrected *P*-values (Benjamini-Hochberg method) for the effect of the interaction between treatment × test period for body mass, CORT and immunological and oxidative stress biomarkers (in the case of PHA skin test, that was only measured at the end of the experiment, parameters for treatment effect alone are reported instead). For the lysis capacity model, we provide odds ratio (OR) and χ2 instead of β and F, respectively. TEAC is computed as residuals of TEAC after controlling by uric acid levels. Full models for each variable are reported in Table S3.

	β/OR	95% CI	F/χ2	df	*P*	Corrected *P*
Body mass (g)	-0.04	-2.04, 1.95	<0.01	1, 56	0.966	0.966
CORT (ng/ml)	-0.03	-0.10, 0.05	0.45	1, 54	0.505	0.505
**Immunity**						
PHA skin test (mm)	-0.12	-0.27, 0.03	2.67	1, 28	0.114	0.460
Total WBC count (cells/10,000 erythrocytes)	-1.80	-10.8, 7.17	0.15	1, 56	0.698	0.924
H:L ratio	-0.09	-0.22, 0.03	2.00	1, 56	0.162	0.460
Lysis (prob)	1.65	0.56, 4.81	0.22	1	0.642	0.924
Agglutination (titers)	0.02	-0.24, 0.27	0.12	1, 50	0.906	0.924
Haptoglobin (mg/ml)	<0.01	-0.04, 0.04	0.01	1, 56	0.924	0.924
Bacteria killing capacity (% *E. coli* dead)	0.41	-0.20, 1.02	1.74	1, 28	0.197	0.460
**Oxidative stress**						
MDA (μmol/L)	-0.12	-0.43, 0.18	0.62	1, 46	0.436	0.751
TEAC (mmol Trolox Eq/L)	0.01	-0.12, 0.14	0.02	1, 36	0.879	0.879
OXY (µmol of HClO neutralized)	0.02	-0.01, 0.06	1.33	1, 36	0.256	0.751
tGSH (mmol/g)	-0.10	-0.37, 0.16	0.58	1, 40	0.451	0.751
GSH:GSSG ratio	-0.38	-2.99, 2.24	0.08	1, 38	0.780	0.879

None of the immunological or oxidative stress markers showed significant effects due to the experimental treatment when comparing LB and HB nestlings (Table 1 and Fig. 2c-n; Table S3). Repeating the MDA analysis while controlling for circulating triglyceride levels still showed a non-significant time × treatment interaction (Table S5). Similar negative results were found when controlling MDA levels by response to PHA and mass gained during the experiment (Table S6).

Results were consistent when considering total begging time as a continuous variable instead of the categorical factor “Treatment” and when analyzing within-dyad differences (LB-HB) in body mass, CORT, and immune and oxidative stress biomarkers as response variables, with the dyad difference in begging as the main predictor (Tables S7-8).

Effect sizes calculated as the β coefficient, Hedges’s g or odds ratio yielded similar conclusions (Table 1; Table S9). All 95% CI of effect sizes included either zero or one (for lysis capacity odds ratio), indicating non-relevant effects for any of the analyzed variables.

## Discussion

Our experimental manipulation significantly increased the begging effort of HB nestlings, who begged 3.7 times longer and with more intense postures than LB nestlings, and 1.7 times longer than hourly averages in wild broods. However, despite such differences in begging effort, we found no significant differences in body mass, CORT levels, immunological markers, or oxidative stress markers between HB and LB nestlings. In all cases, 95% confidence intervals of effect sizes included null values (zero or one for odds ratio in lysis capacity), indicating effects of minimal biological relevance. Our findings of no significant effects contrast with several previous studies. While previous studies on the impact of begging on growth have shown mixed results and evidence for an oxidative cost remains inconclusive (see Introduction), it is notable that we did not observe significant effects on immunological markers either. This contrasts with previous studies that have consistently found an effect, even with lower sample sizes and similar begging schedules (Moreno-Rueda 2010; Moreno-Rueda and Redondo 2011, 2012; Moreno-Rueda et al. 2012; Soler et al. 2014; Redondo et al. 2016). Methodological differences in experimental protocols may explain the discrepancies between our results and prior research.

Some previous studies followed a differential stimulation protocol for HB and LB nestlings (Kilner 2001; Moreno-Rueda 2010; Moreno-Rueda and Redondo 2011, 2012; Moreno-Rueda et al. 2012; Soler et al. 2014; Redondo et al. 2016; Nettle et al. 2017), a common approach in investigating begging costs, albeit with exceptions (Rodríguez-Gironés et al. 2001; Leonard et al. 2003, this study). Typically, HB nestlings receive more frequent stimulation and are often placed in separate environments compared to LB nestlings. For instance, Moreno-Rueda (2010) and Soler et al. (2014) enforced more frequent begging in HB house sparrow (*Passer domesticus*) nestlings, while LB nestlings were stimulated less frequently and fed promptly upon begging initiation. This differential protocol introduces significant stimulation disparities between groups, as discussed in previous research (Casagrande et al. 2016; Redondo et al. 2016). Increased stimulation can induce stress, potentially elevating CORT levels and impacting growth and immune response (Saino et al. 2003). In our experimental design, we mitigated this issue by ensuring both HB and LB nestlings were exposed to similar stimulation: siblings were housed together throughout the experiment, transported together to stimulation trials, and allowed LB nestlings to self-regulate their begging levels. Moreover, unlike previous studies, we confirmed that CORT levels did not influence our findings, as we observed no differences in circulating levels of this mediator of stress responses among treatments.

Many studies advise against protocols that consider only a single or a few isolated markers of immunity or oxidative stress due to the complexity of these systems and recommend incorporating multiple markers instead (Norris and Evans 2000; Keil et al. 2001; Adamo 2004; Halliwell and Gutteridge 2007; Monaghan et al. 2009; Pérez-Rodríguez 2009). This is a limitation in all previous studies assessing the immunological cost of begging, which examined only one or at best two components of the immune system, such as the T-cell induced response to PHA (Moreno-Rueda 2010; Moreno-Rueda and Redondo 2011, 2012; Moreno-Rueda et al. 2012; Soler et al. 2014; Redondo et al. 2016) or inflammatory markers like HSCRP and IL-6 (Nettle et al. 2017). To address this limitation, we tested the effect of begging on seven markers of immune function, thus addressing different immune system components, including cell-mediated, humoral, innate, and acquired responses. We also evaluated whether increased begging effort imposed an oxidative challenge by simultaneously measuring five markers of oxidative damage, antioxidant capacity, and oxidative status. This multi-faceted approach mitigates potential biases from interpreting results based on a single component, especially measures susceptible to observer bias, such as the PHA skin test, which has shown the most consistent results in previous studies. Implementing methods that correct these biases, such as blind measurements, is crucial (Bebarta et al. 2003). Except for Redondo et al. (2016), previous studies using this method did not explicitly state whether patagium thickness was measured blind to chick treatment, posing a potential problem. Animal studies not utilizing blinding methodologies are 3 to 5 times more likely to report differences between study groups than those that do (Bebarta et al. 2003; Wang et al. 2024). In our study, we rigorously adhered to a blind protocol for PHA and also included other physiological variables subject to minimal bias risk.

Our results suggest that when methodological issues from previous studies are addressed, there are no measurable allocation trade-offs between begging and standard markers of nestling health. It must be noted, however, that these trade-offs have been tested under the strict assumptions of Godfray’s differential benefits model (Godfray 1991, 1995). More recent theoretical developments indicate that some of these assumptions are unnecessarily restrictive (Számadó et al. 2019, 2022, 2023; Penn and Számadó 2020). These include:

(i) Direct intrinsic costs are assumed to be independent of offspring nutritional condition. This is likely an unrealistic assumption (Bergstrom et al. 2002) and, when relaxed by incorporating both differential benefits and costs, multiple honest equilibria are possible, including cost-free solutions (Számadó et al. 2019). For example, vigorous begging could interfere with other physiological processes in satiated nestlings, such as postprandial sleep (Spudeit et al. 2013) or intestinal blood flow (Kilner 1997). Alternatively, costs may only be borne by nestlings on a low-quality or restricted diet, as suggested by some studies (Moreno-Rueda and Redondo 2012; Nettle et al. 2017). It follows that any empirical evaluation of costs must relate to the benefits obtained (Moreno-Rueda and Redondo 2012; Számadó et al. 2019).

(ii) Signal cost as a function of signal intensity increases monotonically. If the cost of a signal rises sharply at the point where it would be dishonest, signaling below that point can be cheap or free (Lachmann et al. 2001; Bergstrom et al. 2002). Costs might only be detectable after forcing chicks to beg at deceptive (out-of-equilibrium) levels (Higham 2014), which is challenging to implement empirically, as satiated chicks do not beg voluntarily, and artificial trait manipulations can result in artifacts (Kotiaho 2000). For example, histamine agonists may enhance nestling begging (Martín-Gálvez et al. 2011), but they also interfere with normal immune responses (Beer et al. 1984).

(iii) At the honest equilibrium, offspring in prime condition produce no signals and incur no signal costs. When this constraint is relaxed, physiological trade-offs may only be measurable above a certain threshold of signal intensity, allowing for minimal or zero-cost signals except at the highest signal levels (Bergstrom and Lachmann 1998; Lachmann and Bergstrom 1998). Although no study has yet addressed this problem, LB begging rates in our study were higher than those in 83% of previous studies (Soler et al. 2014; Nettle et al. 2017) and within the natural range of variation, potentially offering a more realistic estimation of marginal costs.

Other assumptions of costly signaling models of begging have also been questioned, suggesting the possibility of honest yet minimal or cost-free scenarios (Számadó 2011; Zollman 2013). First, costs may diminish or vanish when genetic relatedness is adequately incorporated into fitness calculations (Bruner and Rubin 2020). Second, most models assume begging is highly informative and requires substantial direct costs to maintain honesty (Brilot and Johnstone 2002, 2003). This assumption has not been critically tested, but constraints on both signalers and receivers (Broom and Ruxton 2013; Peniston et al. 2020) might promote the evolution of honest yet partially informative communication between relatives allowing for low or cost-free equilibria (Bergstrom and Lachmann 1998; Lachmann and Bergstrom 1998; Zollman 2013; Zollman et al. 2013). Finally, selective parental inattention (Rich and Zollman 2016; Whitmeyer 2021) may reduce the incentive for deceit, decreasing the need for a stabilizing direct trade-off. While costly signaling models typically assume parents always respond positively to extravagant begging, observations of parental behavior suggest otherwise (Estramil et al. 2013; Parejo-Pulido et al. 2023), possibly because parents use additional sources of information about offspring condition (Rodríguez-Gironés et al. 1996; Mock et al. 2011).

Even without evidence that escalated begging harms nestling health, our results align with the trade-off signaling model (Számadó et al. 2019, 2023), which suggests that the measured cost at equilibrium could be zero but contradict traditional “costly signaling” models (Grafen 1990; Godfray 1991) that predict an equilibrium cost. However, a comprehensive test of the trade-off model must consider the differential benefits of escalated begging, particularly that the marginal benefit of an out-of-equilibrium signal should be smaller than its marginal cost (Számadó et al. 2019, 2022). This prediction also corresponds with previous findings that hungrier nestlings, who gain more from additional food (Grodzinski et al. 2011), beg more vigorously and receive greater parental provisioning (Searcy and Nowicki 2005). Finally, our study does not explore other potential trade-offs in begging, such as the possibility that escalated begging may reduce the viability of other family members or increase predation risk.

In conclusion, supporting evidence for physiological trade-offs in nestling begging displays, as predicted by costly signaling models, is inconsistent and often limited by methodological concerns. Our study shows that, after addressing these issues, there are no measurable trade-offs between begging and multiple standard markers of nestling health (growth, immune function, and oxidative status). However, these trade-offs have been tested under the strict assumptions of a single, albeit highly influential, theoretical model, which may be unnecessarily restrictive. Further theoretical developments suggest that assumptions about direct intrinsic costs, signal cost functions, and honest equilibrium constraints may need revision.

## Supplementary Material

araf003_suppl_Supplementary_Tables_S1-S9

## Data Availability

Analyses reported in this article can be reproduced using the data providedby Parejo-Pulido et al. (2025).
