# Supplementary material for: Escalated begging does not compromise nestling health
Source: Behav Ecol. 2025 Jan 28;36(2):araf003. doi: 10.1093/beheco/araf003 (PMC12257937; doi:10.1093/beheco/araf003)
Supplement: araf003_suppl_Supplementary_Tables_S1-S9 [file araf003_suppl_supplementary_tables_s1-s9.pdf]

SUPPLEMENTARY DATA

Title: Escalated begging does not compromise nestling health

Short title: Begging does not compromise health

Daniel Parejo-Pulido[1], Tomás Redondo[2], Silvia Casquero[1] and Lorenzo Pérez-Rodríguez[1]

[1]Instituto de Investigación en Recursos Cinegéticos (IREC), CSIC-UCLM-JCCM, Ronda de Toledo 12, 13005 Ciudad Real, Spain

[2]Estación Biológica de Doñana (EBD), CSIC, Américo Vespucio 26, 41092 Sevilla, Spain

Corresponding author: Daniel Parejo-Pulido. Instituto de Investigación en Recursos Cinegéticos (IREC), CSIC-UCLM-JCCM, Ronda de Toledo 12, 13005 Ciudad Real, Spain. +34 615874731. dapapu96@gmail.com

ORCID: Daniel Parejo-Pulido: https://orcid.org/0000-0002-7392-2423; Tomás Redondo: https://orcid.org/0000-0002-0927-9111; Silvia Casquero: https://orcid.org/0000-0002-5466-7770; Lorenzo Pérez-Rodríguez: https://orcid.org/0000-0002-5926-1438

IMMUNOLOGICAL AND OXIDATIVE ASSAYS

PHA skin test

PHA-P is a protein that induces an inflammatory swelling of the patagium skin, which correlates with the strength of the T cell–mediated immune response (Kennedy and Nager 2006), though other immune system components are also involved (Martin et al. 2006). We followed the simplified protocol by Smits et al. (1999). On the morning of day 10, immediately after the first begging session (08:30 h), we measured the left wing patagium thickness of each nestling three times using a pressure-sensitive micrometer (Mitutoyo; accuracy: 0.01 mm) and injected 0.2 mg of phytohemagglutinin (PHA-P, L-8754, Sigma–Aldrich) diluted in 0.04 ml of isotonic phosphate buffer. At the end of the day, after the last begging session (20:30 h, 12 h later), we measured the patagium thickness again. We calculated the T cell–mediated immune response as the difference between initial and final measurements. The repeatability of the initial and final measurements was high (ICC = 0.955, N = 58, $F_{5,57}$ = 178, P < 0.001; ICC = 0.984, N = 58, $F_{4,57}$ = 708, P < 0.001, respectively).

White blood cells count

Before centrifuging blood samples, we smeared a drop of blood onto marked microscope slides, air-dried them, fixed them in methanol, and stained them using a Quick Panoptic kit (Ref. 993090, Química Clínica Aplicada S.A.). We examined the blood smears under a microscope (Nikon ECLIPSE Ci-L) with a Digital Color Camera (Nikon DS Ri1) to obtain two measures: (1) total white blood cell (WBC) count and (2) differential WBC count. For the total WBC count, we captured images of 70 consecutive homogeneous fields of view (40x magnification) per sample and counted the WBCs. We then used Mizutama (Ochoa et al. 2019) to count erythrocytes in each image (Parameter settings: Contrast = 0.4, Gray threshold = 0.5, Diameter = 0.04, Fraction = 0.3, and Gray fraction = 0.4). We calculated WBC count as the number of cells per 10,000

erythrocytes (Muriel et al. 2021). For the differential WBC count, we examined the smears using the oil immersion objective (1000x magnification) to estimate the proportion of different WBC types over a total of 100 WBCs (Campbell and Ellis 2007). WBCs were classified as heterophils, eosinophils, basophils, lymphocytes, or monocytes. From these proportions, we calculated the heterophil/lymphocyte (H:L) ratio used in the analyses.

Lysis and agglutination assay

We quantified natural antibody titers (agglutination titer) and complement activity (lysis titer) using the lysis and agglutination assay, with higher levels indicating better immune function (Matson et al. 2005). We followed the protocol by Matson et al. (2005) with minor modifications. We added 25 µl of each plasma sample to the first and second rows of U-shaped 96-well plates. Samples were serially diluted (1:2) from rows 2-8 by adding 25 µl of 0.01M phosphate-buffered saline (PBS). Then, we added 25 µl of a 0.1% fresh sheep red blood cells suspension to all wells, gently vortexed the plates, and incubated them at 37°C for 90 minutes. After incubation, plates were tilted at 45° at room temperature for 20 minutes before scoring lysis and agglutination titers using a stereo microscope. Agglutination was identified by clumped red blood cells due to antibodies binding multiple antigens, while lysis was evident as the destruction of all red blood cells in the well. Titers were recorded as the negative log2 of the last plasma dilution showing positive agglutination or lysis. Due to limitations in plasma availability, we assessed repeatability using a different set of 22 adult spotless starling samples (lysis: ICC = 0.895, $F_{22,21}$ = 17.8, P < 0.001; agglutination: ICC = 0.903, $F_{22,21}$ = 19.9, P < 0.001).

Haptoglobin levels

Haptoglobin is an acute phase protein that increases rapidly in response to infection, inflammation, or trauma (Millet et al. 2007). We measured haptoglobin levels in nestling plasma samples using a commercial colorimetric kit (TP801, Tridelta Development Ltd.) following the

manufacturer's instructions with minor modifications (Matson et al. 2012). In flat-bottom 96-well plates, we added 6 µl of plasma sample or standard and 80 µl of reagent 1. After a brief vortex, we recorded absorbances at 450 and 630 nm using a Thermo Scientific Multiskan GO microplate reader. We then added 112 µl of reagent 2, incubated the plates at 22°C for 5 minutes, and took a final absorbance measurement at 630 nm. We subtracted the initial 630 nm absorbance (before adding reagent 2) from the post-incubation measurements to correct for plasma color and turbidity artifacts. The 450 nm pre-scan enabled us to statistically analyse and correct for differences in plasma sample redness, indicating sample haemolysis, which can affect assay results (Matson et al. 2012). Since this value did not significantly affect haptoglobin concentration in our samples (P = 0.704), we excluded it from the final models to avoid model saturation. We calculated haptoglobin concentrations (mg/ml) using the standard curve on each plate. We assessed the repeatability of this technique using a different set of samples from 9-day-old spotless starling nestlings from 2022 (ICC = 0.703, N = 15, $F_{14,14}$ = 5.42, P = 0.002).

Bacterial killing capacity

The bacterial killing capacity assay measures the innate immune ability of plasma to lyse *Escherichia coli*, with increased killing capacity indicating stronger immune function (Matson et al. 2006). We grew *E. coli* in LB medium and counted the bacteria using a MACSQuant X cytometer (Miltenyibiotec), which provides absolute counts via a syringe delivery system. The bacteria culture was then diluted in PBS in 96-well plates to 10 million bacteria per well in 100 µl. We added 5 µl of each plasma sample to the wells and incubated them for 24 hours at 30 °C. Controls included the same amount of PBS diluent. After incubation, plates were treated with the Life & Death kit (Thermofisher) reagent, containing Propidium Iodide (PI) to detect dead cells and Syto 14 as a total cell marker, for 15 minutes at culture temperature. We analysed the plates using the same cytometer, quantifying PI-positive (dead) cells. The analysis was performed with

Flowlogic software (Inivai Technologies). We calculated the percentage of dead *E. coli* cells relative to the initial concentration for the analyses. Samples were measured in duplicate and showed high repeatability (ICC = 0.978, N = 60, $F5_{9,59}$ = 89.1, $P < 0.001$).

MDA

We measured plasma levels of malondialdehyde (MDA), a widely used marker of oxidative damage to lipids (Del Rio et al. 2005; Mateos and Bravo 2007) and a proxy for whole-body oxidative damage (Argüelles et al. 2004; Margaritelis et al. 2015). We quantified MDA using high-performance liquid chromatography (HPLC) following published protocols (Agarwal and Chase 2002; Romero-Haro and Alonso-Alvarez 2014). Briefly, we added 50 µl of butylated hydroxytoluene (0.05% w/v in 95% ethanol), 400 µl of 0.44 M phosphoric acid, 100 µl of 42 mM thiobarbituric acid (TBA), and 30 µl of distilled water to 20 µl of each plasma sample. The mixture was vortexed and incubated at 100 °C for 1 hour to form MDA-TBA adducts. We then cooled the tubes on ice for 5 minutes, added 250 µl of n-butanol, vortexed for 60 seconds, and centrifuged at 18,000 g at 4 °C for 3 minutes. The upper phase was transferred to amber chromatography vials and injected into an Agilent 1200 series HPLC system with a fluorescence detector set at 515 nm (excitation) and 553 nm (emission). We used a C18 column (5-mm ODS-2 4.0 x 250-mm) at 37 °C and an isocratic flow of MeOH:KH2PO4 (50 mM; 40:60 v/v) at 1 ml/min. We used an 8-point serial dilution of 10 µM 1,1,3,3-tetraethoxypropane as the calibration curve. Data are reported in µmoles of MDA per L of plasma. Repeatability of this technique was validated using a different set of samples from 6-day-old spotless starling nestlings in 2021 (ICC = 0.800, N = 18, $F_{17,17}$ = 8.61, $P < 0.001$).

Trolox Equivalent Antioxidant Capacity (TEAC) assay

We estimated the combined action of nonenzymatic antioxidants in plasma using the TEAC assay, following Miller et al. (1993), with modifications by Cohen et al. (2007) (see also López-

Arrabé et al. 2015). This assay is commercially known as Total Antioxidant Status (TAS). We used flat-bottom 96-well microplates, arranging plasma samples (5 µl) in rows and mixing them with 15 µl of 153 µM metmyoglobin and 250 µl of the chromogen ABTS (2,2-azino-di-[3-ethylbenzthiazolinesulphonate]). Each row included two wells with 5 µl duplicates of the standard, a 1.7 mM water-soluble α-tocopherol derivative (Trolox). We initiated the reaction by adding 50 µl of 600 µM hydrogen peroxide to all wells and monitored absorbance changes at 660 nm every 10 seconds for 10 minutes at 37 °C using the microplate reader. We recorded the time at which absorbance increased, indicating the sample no longer protected the chromogen from oxidation. We calculated antioxidant capacity relative to the average values of the Trolox standards in the same row, reported as mmoles of Trolox equivalents per litre of plasma. We assayed samples in duplicate, achieving high repeatability (ICC = 0.895, N = 70, $F_{69,69} = 17.8$, $P < 0.001$).

Antioxidant capacity of plasma (OXY)

We evaluated the antioxidant capacity of plasma samples using the OXY-adsorbent Assay (Diacron, Grosetto, Italy) following the manufacturer's instructions with minor modifications for small volumes and a microplate reader. Each plasma sample was diluted 1:100 in ultrapure distilled water, and 10 µL of each diluted sample was pipetted into a well of a flat-bottom 96-well microplate. We also pipetted 10 µL of the kit calibrator and blank (ultrapure water) in duplicate with each batch of samples. We then added 150 µL of HClO solution to each well, briefly vortexed (5 s), and measured absorbances at 546 nm with the microplate reader. This initial absorbance was subtracted from the final measurements to control for sample turbidity. The plate was incubated for 10 minutes at 37 °C. After incubation, we added 50 µL of chromogen (diluted 1:10 in ultrapure water), which reacts with the residual HClO not neutralized by antioxidants in the sample, forming a pink derivative. The intensity of the coloured complex,

inversely related to the antioxidant power of the sample, was measured at 546 nm immediately after adding the chromogen. Measurements are expressed as µmoles of HClO neutralized. We confirmed the repeatability of this technique using a different set of samples from nestlings of similar age to those used in this experiment (ICC = 0.959, N = 18, $F_{17,15}$ = 54.5, P < 0.001).

tGSH and GSH:GSSG ratio

Total levels of glutathione (tGSH) and the ratio of reduced to oxidized glutathione (GSH:GSSG ratio) represent the functional levels of a key intracellular antioxidant and the overall redox state of the cell (Halliwell and Gutteridge 2007). For its quantification in erythrocytes, we followed Griffith (1980) with modifications (Romero-Haro and Alonso-Alvarez 2014). Washed erythrocytes were diluted (1:10 w/v) and homogenized in a stock buffer (PBS and 0.02 M EDTA). An aliquot (0.5 ml) of homogenate was vortexed with 0.5 ml of 10% trichloroacetic acid three times for 5 s each over 15 min. The mixture was centrifuged (1,125 g for 15 min at 6 °C), and the supernatant was removed. An automated spectrophotometer (A25 Biosystems autoanalyzer) was used for subsequent steps. We prepared three solutions in the stock buffer: 0.3 mM NADPH (solution 1), 6 mM DTNB (solution 2), and 50 U/mL GSH reductase (solution 3). Solutions 1 and 2 were mixed (7:1 v/v), and 160 µl of this mixture was added to 40 µl of the sample in a cuvette. After 15 s, 20 µl of solution 3 was added, and absorbance at 405 nm was monitored at 30 and 60 s. We determined tGSH levels by comparing absorbance changes to a GSH standard curve (1 to 0.031 mM) and expressed results in µmol per gram of erythrocytes.

For GSSG, an aliquot (400 µl) of the tGSH supernatant was adjusted to pH 7.5 with 6 N NaOH, then 8 µl of 2-vinylpyridine was added. The mixture was shaken at room temperature in the dark, then centrifuged (1,125 g for 10 min). Absorbance changes at 405 nm were measured as described for tGSH. Reduced GSH was calculated by subtracting GSSG from tGSH, and the GSH:GSSG ratio was then determined (Owen and Butterfield 2010). We assessed repeatability

with samples assayed in duplicate (tGSH: ICC = 0.961, N = 143, $F_{142,142}$ = 50.8, $P < 0.001$; GSSG: ICC = 0.975, N = 141, $F_{140,140}$ = 77.6, $P < 0.001$).

Triglycerides and Uric acid

Both MDA and TEAC values are highly sensitive to plasma triglycerides and uric acid levels, respectively, making advisable to statistically control for them in analyses (Cohen et al. 2007; Pérez-Rodríguez et al. 2015). We quantified these metabolites using commercial kits (refs. 11,522 and 11,529; Biosystems, Barcelona, Spain) based on the glycerol phosphate oxidase/peroxidase (for triglycerides) and uricase/peroxidase (for uric acid) methods. We followed the manufacturer’s instructions, with minor modifications for sample, calibrator, and reagent volumes in flat-bottom 96-well microplates, adding the kit reagent and measuring absorbances at the specified wavelength with the same reader mentioned earlier. To control for sample turbidity's potential impact on absorbances, we performed the same procedure using ultrapure water instead of the reagent for all samples and calibrators. We subtracted the absorbance of these “blank” measurements from all samples before calculating final concentrations, expressed in mg per dL of plasma for both metabolites. A subset of samples, assayed in duplicate, showed high repeatability for triglyceride (ICC = 0.996, N = 16, $F_{15,15}$ = 534, $P < 0.001$) and for uric acid levels (ICC = 0.995, N = 24, $F_{23,23}$ = 412, $P < 0.001$).

Corticosterone

We quantified plasma corticosterone (CORT) levels following the protocol from (Gil et al. 2019) with minor modifications. We added 1 ml of extra pure diethyl ether to 15 µl of plasma, vortexed for 2 min, and centrifuged for 10 min (1.4 rcf). Tubes were then frozen for 3 min in a dry ice/96% ethanol bath. The upper phase was transferred to clean tubes and dried in a heated block for 10-15 min. Extracts were resuspended in 200 µl of steroid-free serum. Most samples were assayed in duplicate using a commercial ELISA kit (Cat No. K014-H1/H5, Arbor Assays, DetectX®)

and the microplate reader mentioned earlier. Repeatability of samples assayed in duplicate was high (ICC = 0.998, N = 99, $F_{98,98}$ = 1062, P < 0.001).

Additional statistical analyses

We confirmed the robustness of our main results using additional analytical approaches. First, we checked differences in MDA levels according to treatment and time, considering the response to PHA and mass gain as covariates, as done in Moreno-Rueda et al. (2012) (Supplementary Table S6). Second, to ensure that the "Treatment" factor does not obscure individual variability in begging performance within each level, we repeated the analyses using a continuous variable: average begging effort per nestling. We reported only results using time begging as a predictor, since other measures yielded the same results. For each nestling, we calculated predicted begging time at a specific point (trial = 2.5, session = 1.5, day = 1.5) to derive an average value for the four recorded sessions. In these models, the response variables were the post-treatment measures of each physiological variable (body mass, immunological, and oxidative markers), controlled for initial levels and mass as covariates (Supplementary Table S7). For MDA and TEAC analyses, initial levels were computed as residuals of initial values controlled for triglyceride and uric acid levels, respectively. We included the nest of origin nested within the date of arrival at the laboratory as random effects. For CORT, MDA, and TEAC analyses, we also included handling time, triglyceride, and uric acid levels of the final measure, respectively.

Additionally, we checked for unequal differences in begging effort of HB and LB nestlings between dyads. This approach is similar to that used by Kilner (2001) and Leonard et al. (2003) in measuring growth costs of begging in canaries and barn swallows. To address this, we repeated models using the difference HB-LB in final measures as the response variable, controlling for differences in initial levels, time begging, and initial body mass (Within-Dyad differences models). For MDA and TEAC analyses, differences in initial levels were computed as

residuals controlling for triglyceride and uric acid levels. For CORT, MDA, and TAS analyses, we included differences in handling time, triglyceride, and uric acid levels of the final measure. The date of arrival at the laboratory was included as a random effect.

REFERENCES

Agarwal R, Chase SD. 2002. Rapid, fluorimetric–liquid chromatographic determination of malondialdehyde in biological samples. J Chromatogr B. 775(1):121–126.

Argüelles S, García S, Maldonado M, Machado A, Ayala A. 2004. Do the serum oxidative stress biomarkers provide a reasonable index of the general oxidative stress status? Biochim Biophys Acta Gen Subj. 1674(3):251–259.

Campbell TW, Ellis CK. 2007. Avian and Exotic Animal Hematology and Cytology. Ames: Blackwell Publishing Professional.

Cohen A, Klasing K, Ricklefs R. 2007. Measuring circulating antioxidants in wild birds. Comp Biochem Physiol B Biochem Mol Biol. 147(1):110–121.

Gil D, Alfonso-Iñiguez S, Pérez-Rodríguez L, Muriel J, Monclús R. 2019. Harsh conditions during early development influence telomere length in an altricial passerine: Links with oxidative stress and corticosteroids. J Evol Biol. 32(1):111–125.

Griffith OW. 1980. Determination of glutathione and glutathione disulfide using glutathione reductase and 2-vinylpyridine. Anal Biochem. 106(1):207–212.

Halliwell B, Gutteridge JMC. 2007. Free radicals in biology and medicine. Oxford: Oxford University Press.

Kennedy MW, Nager RG. 2006. The perils and prospects of using phytohaemagglutinin in evolutionary ecology. Trends Ecol Evol. 21(12):653–655.

Kilner RM. 2001. A growth cost of begging in captive canary chicks. Proc Natl Acad Sci USA. 98(20):11394–11398.

Leonard ML, Horn AG, Porter J. 2003. Does begging affect growth in nestling tree swallows, *Tachycineta bicolor*? Behav Ecol Sociobiol. 54(6):573–577.

López-Arrabé J, Cantarero A, Pérez-Rodríguez L, Palma A, Alonso-Alvarez C, González-Braojos S, Moreno J. 2015. Nest-dwelling ectoparasites reduce antioxidant defences in females and nestlings of a passerine: a field experiment. Oecologia. 179(1):29–41.

Margaritelis N V., Veskoukis AS, Paschalis V, Vrabas IS, Dipla K, Zafeiridis A, Kyparos A, Nikolaidis MG. 2015. Blood reflects tissue oxidative stress: a systematic review. Biomarkers. 20(2):97–108.

Martin LB, Han P, Lewittes J, Kuhlman JR, Klasing KC, Wikelski M. 2006. Phytohemagglutinin-induced skin swelling in birds: histological support for a classic immunoecological technique. Funct Ecol. 20(2):290–299.

Mateos R, Bravo L. 2007. Chromatographic and electrophoretic methods for the analysis of biomarkers of oxidative damage to macromolecules (DNA, lipids, and proteins). J Sep Sci. 30(2):175–191.

Matson KD, Horrocks NPC, Versteegh MA, Tieleman BI. 2012. Baseline haptoglobin concentrations are repeatable and predictive of certain aspects of a subsequent experimentally-induced inflammatory response. Comp Biochem Physiol A Mol Integr Physiol. 162(1):7–15.

Matson KD, Ricklefs RE, Klasing KC. 2005. A hemolysis-hemagglutination assay for characterizing constitutive innate humoral immunity in wild and domestic birds. Dev Comp Immunol. 29(3):275–286.

Matson KD, Tieleman BI, Klasing KC. 2006. Capture stress and the bactericidal competence of blood and plasma in five species of tropical birds. Physiol Biochem Zool. 79(3):556–564.

Miller NJ, Rice-Evans C, Davies MJ, Gopinathan V, Milner A. 1993. A novel method for measuring antioxidant capacity and its application to monitoring the antioxidant status in premature neonates. Clin Sci. 84(4):407–412.

Millet S, Bennett J, Lee KA, Hau M, Klasing KC. 2007. Quantifying and comparing constitutive immunity across avian species. Dev Comp Immunol. 31(2):188–201.

Moreno-Rueda G, Redondo T, Trenzado CE, Sanz A, Zúñiga JM. 2012. Oxidative stress mediates physiological costs of begging in magpie (*Pica pica*) nestlings. PLoS One. 7(7):e40367.

Muriel J, Vida C, Gil D, Pérez-Rodríguez L. 2021. Ontogeny of leukocyte profiles in a wild altricial passerine. J Comp Physiol B. 191(1):195–206.

Ochoa D, Redondo T, Moreno-Rueda G. 2019. Mizutama: A Quick, Easy, and Accurate Method for Counting Erythrocytes. Physiol Biochem Zool. 92(2):206–210.

Owen J, Butterfield D. 2010. Measurement of oxidized/reduced glutathione ratio. In: Bross P, Gregersen N, editors. Protein misfolding and cellular stress in diseases and aging: concepts and protocols. Vol. 648 of Methods in Molecular Biology. New York, NY: Springer. p. 269–277.

Pérez-Rodríguez L, Romero-Haro AA, Sternalski A, Muriel J, Mougeot F, Gil D, Alonso-Alvarez C. 2015. Measuring oxidative stress: the confounding effect of lipid concentration in measures of lipid peroxidation. Physiol Biochem Zool. 88(3):345–351.

Del Rio D, Stewart AJ, Pellegrini N. 2005. A review of recent studies on malondialdehyde as toxic molecule and biological marker of oxidative stress. Nutr Metab Cardiovasc Dis. 15(4):316–328.

Romero-Haro AA, Alonso-Alvarez C. 2014. Covariation in oxidative stress markers in the blood of nestling and adult birds. Physiol Biochem Zool. 87(2):353–362.

Smits JE, Bortolotti GR, Tella JL. 1999. Simplifying the phytohaemagglutinin skin-testing technique in studies of avian immunocompetence. Funct Ecol. 13(4):567–572.

TABLE S1: Mean ± SE initial values of body mass, CORT levels, immunological and oxidative stress markers measured in this study for low-begging (LB) and high-begging (HB) experimental groups. We also provide F values, df, uncorrected and corrected *P*-values (Benjamini-Hochberg method) for the effect of the treatment. For generalized linear mixed models analyzing lysis capacity we provide χ2 instead of F. TEAC is computed as residuals of TEAC after controlling by uric acid levels.

	LB	HB	F/ χ2	df	*P*	Corrected *P*
**Initial body mass (g)**	48.6 ± 1.27	48.7 ± 1.18	<0.01	1, 28	0.967	0.967
**CORT (ng/ml)**	0.53 ± 0.12	0.43 ± 0.07	0.07	1, 26	0.796	0.796
**Immunological assays**						
Total WBC count (cells/10,000 erythrocytes)	24.1 ± 2.66	24.2 ± 1.62	<0.01	1, 28	0.966	0.966
H:L ratio	0.92 ± 0.12	1.03 ± 0.13	0.58	1, 28	0.455	0.893
Lysis (prob)	0.23 ± 0.08	0.27 ± 0.09	0.17	1	0.679	0.893
Agglutination (titers)	1.04 ± 0.12	1.00 ± 0.14	0.11	1, 25	0.745	0.893
Haptoglobin (mg/ml)	0.06 ± 0.01	0.07 ± 0.02	0.42	1, 28	0.522	0.893
Bacterial killing capacity (% *E. coli* dead)	21.2 ± 5.32	19.0 ± 4.77	4.29	1, 13	0.058	0.460
**Oxidative stress**						
MDA (µmol/L)	1.73 ± 0.12	1.63 ± 0.12	2.37	1, 22	0.137	0.687
TEAC (residuals) (mmol Trolox Eq/L)	0.06 ± 0.03	0.06 ± 0.04	0.12	1, 17	0.735	0.735
OXY (µmol of HClO neutralized)	348 ± 2.56	344 ± 4.47	0.59	1, 18	0.453	0.735
tGSH (mmol/g)	2.81 ± 0.16	2.85 ± 0.11	0.15	1, 19	0.699	0.735
GSH:GSSG ratio	8.52 ± 0.77	7.99 ± 0.82	0.36	1, 19	0.555	0.735

TABLE S2: Estimates (± SE), *t* values and *P*-values for models explaining differences in consumed food (total grams of boiled egg and crickets) and begging effort (time begging, begging score, mean postural intensity and time begging at intensity levels 4 and 5). We also provide corrected *P*-values (Benjamini-Hochberg method) for the effect of treatment (LB or HB). "*Treatment*" is a categorical factor with "*LB*" as the reference group.

	**Estimate**	**SE**	**t**	***P***	**Corrected *P***
**Consumed food (g)**					
Intercept	49.5	0.45	111	<0.001	
Treatment	-0.46	0.29	-1.58	0.126	0.126
Initial mass	3.58	0.29	12.3	<0.001	
**Time begging (s/trial)**					
Intercept	9.37	0.73	12.9	<0.001	
Treatment	24.9	0.98	25.5	<0.001	<0.001
Initial mass	1.43	0.54	2.68	0.012	
Day	0.93	0.17	5.59	<0.001	
Session	0.85	0.17	5.10	<0.001	
Trial	0.26	0.17	1.56	0.118	
**Begging score**					
Intercept	20.7	2.92	7.06	<0.001	
Treatment	84.3	3.06	27.6	<0.001	<0.001
Initial mass	-0.36	2.43	-0.15	0.882	
Day	4.45	0.59	7.52	<0.001	
Session	5.85	0.59	9.88	<0.001	
Trial	-0.03	0.59	-0.05	0.959	
**Mean postural intensity**					
Intercept	2.18	0.10	22.1	<0.001	
Treatment	0.91	0.09	9.73	<0.001	<0.001
Initial mass	-0.14	0.08	-1.70	0.098	
Day	0.07	0.02	3.41	0.001	
Session	0.13	0.02	6.83	<0.001	
Trial	-0.14	0.02	-7.09	<0.001	
**Time begging at levels 4+5**					
Intercept	1.15	0.90	1.28	0.211	
Treatment	11.1	1.15	9.57	<0.001	<0.001
Initial mass	-1.29	0.70	-1.86	0.073	
Day	0.59	0.18	3.31	0.001	
Session	1.46	0.18	8.18	<0.001	
Trial	-0.14	0.18	-0.76	0.447	

TABLE S3: Estimates (± SE), *t* values (z values for total WBC count and lysis analyses) and *P*-values for models explaining final mass, CORT levels, immunological and oxidative stress markers among LB and HB nestlings. We also provide corrected *P*-values (Benjamini-Hochberg method) for the effect of the experimental treatment (LB or HB) in growth and PHA analyses and for the interactions involving the treatment and time of measurement (initial and final). "*Treatment*" and "*Time*" are categorical factors with "*LB*" and "*initial levels*" as reference groups.

	Estimate	SE	t/z	*P*	Corrected *P*
**Growth**					
**Body mass**					
Intercept	48.6	1.28	37.9	<0.001	
Treatment × Time	-0.04	1.02	-0.04	0.966	0.966
Treatment	0.03	0.82	0.03	0.974	
Time	15.5	0.72	21.5	<0.001	
**Body mass day 9**					
Intercept	46.4	1.17	39.6	<0.001	
Treatment × Time	-0.03	0.52	-0.05	0.958	0.966
Treatment	-0.09	0.69	-0.13	0.898	
Time	11.2	0.37	30.6	<0.001	
**Body mass day 10**					
Intercept	52.6	1.21	43.6	<0.001	
Treatment × Time	0.27	0.45	0.61	0.545	0.966
Treatment	-0.29	0.70	-0.42	0.680	
Time	11.5	0.32	36.4	<0.001	
**CORT**					
Intercept	0.23	0.02	11.0	<0.001	
Treatment × Time	-0.03	0.04	-0.67	0.505	0.505
Treatment	-0.01	0.03	-0.28	0.782	
Time	-0.01	0.03	-0.37	0.710	
Initial mass	-0.03	0.01	-2.91	0.007	
Handling time	0.02	0.01	1.65	0.103	
**Immunity**					
**PHA skin test**					
Intercept	1.57	0.07	22.9	<0.001	
Treatment	-0.12	0.07	-1.63	0.114	0.460
Initial mass	0.01	0.06	0.25	0.801	
**Total WBC count**					
Intercept	24.2	2.72	8.88	<0.001	
Treatment × Time	-1.80	4.61	-0.39	0.698	0.924

Treatment	0.13	3.26	0.04	0.970	
Time	0.70	3.26	0.21	0.832	
Initial mass	0.90	1.70	0.53	0.599	
**H:L ratio**					
Intercept	0.47	0.04	13.2	<0.001	
Treatment × Time	-0.09	0.07	-1.42	0.162	0.460
Treatment	0.03	0.05	0.61	0.547	
Time	0.10	0.05	2.17	0.035	
Initial mass	-0.05	0.02	-2.63	0.013	
**Lysis (prob)**					
Intercept	-1.98	0.80	-2.48	0.013	
Treatment × Time	0.50	1.07	0.47	0.642	0.924
Treatment	0.30	0.78	0.39	0.696	
Time	0.30	0.78	0.39	0.699	
Initial mass	-0.06	0.45	-0.13	0.897	
**Agglutination**					
Intercept	0.67	0.07	9.69	<0.001	
Treatment × Time	0.02	0.13	0.12	0.906	0.924
Treatment	-0.03	0.09	-0.35	0.725	
Time	0.21	0.09	2.23	0.030	
Initial mass	0.03	0.04	0.69	0.495	
**Haptoglobin**					
Intercept	0.05	0.01	3.45	0.001	
Treatment × Time	<0.01	0.02	0.10	0.924	0.924
Treatment	0.01	0.02	0.47	0.642	
Time	0.08	0.02	5.22	<0.001	
Initial mass	-0.01	0.01	-1.48	0.148	
**Bacterial killing capacity**					
Intercept	-1.71	0.41	-4.18	0.001	
Treatment × Time	0.41	0.31	1.32	0.197	0.460
Treatment	-0.15	0.24	-0.65	0.519	
Time	-0.01	0.22	-0.07	0.947	
Initial mass	0.19	0.23	0.84	0.414	
**Oxidative stress**					
**MDA**					
Intercept	1.76	0.16	11.1	<0.001	
Treatment × Time	-0.12	0.16	-0.79	0.436	0.751
Treatment	-0.10	0.11	-0.92	0.360	
Time	0.51	0.11	4.61	<0.001	
Initial mass	-0.01	0.09	-0.16	0.872	
**TEAC**					
Intercept	0.84	0.04	22.1	<0.001	
Treatment × Time	0.01	0.07	0.15	0.879	0.879

Treatment	<0.01	0.05	0.02	0.983	
Time	-0.13	0.05	-2.73	0.010	
Initial mass	0.03	0.02	1.30	0.216	
Uric acid levels	0.20	0.02	9.19	<0.001	
**OXY**					
Intercept	5.85	0.01	611	<0.001	
Treatment × Time	0.02	0.02	1.15	0.256	0.751
Treatment	-0.01	0.01	-0.97	0.337	
Time	0.04	0.01	2.70	0.011	
Initial mass	<0.01	0.01	0.32	0.756	
**tGSH**					
Intercept	2.81	0.13	22.3	<0.001	
Treatment × Time	-0.10	0.13	-0.76	0.451	0.751
Treatment	0.04	0.11	0.34	0.738	
Time	-0.08	0.10	-0.84	0.404	
Initial mass	0.04	0.09	0.38	0.703	
**GSH:GSSG ratio**					
Intercept	8.53	0.81	10.6	<0.001	
Treatment × Time	-0.38	1.35	-0.28	0.780	0.879
Treatment	-0.53	0.95	-0.56	0.580	
Time	-0.17	0.95	-0.17	0.863	
Initial mass	0.07	0.52	0.14	0.889	

TABLE S4: Estimates (± SE), *t* values and *P*-values for the model explaining differences in CORT levels between experimental nestlings (either LB or HB) and unmanipulated nestlings from natural nests. "*Group*" is a categorical factor with "*Experimental nestlings*" as the reference group.

	**Estimate**	**SE**	**t**	***P***
Intercept	0.19	0.02	11.9	<0.001
Group	<0.01	0.03	0.16	0.877
Body mass	-0.04	0.01	-2.65	0.012
Handling time	<0.01	0.01	0.22	0.828

TABLE S5: Estimates (± SE), *t* values and *P*-values for the three alternative models explaining differences in MDA levels but incorporating plasma triglyceride levels as covariate. We also provide the corrected *P*-values (Benjamini-Hochberg method) for the interaction involving the treatment and time of measurement (initial or final), the time begging and the difference between HB and LB nestlings in time begging. "*Treatment*" and "*Time*" are categorical factors with "*LB*" and "*initial levels*" as reference groups. Initial levels of MDA were computed as residuals of MDA corrected by triglyceride levels.

	**Estimate**	**SE**	**t**	***P***	**Corrected *P***
**Repeated measurements model**					
Intercept	1.71	0.17	10.3	<0.001	
Treatment × Time	-0.18	0.16	-1.17	0.251	0.641
Treatment	-0.06	0.11	-0.53	0.596	
Time	0.36	0.11	3.19	0.003	
Initial mass	-0.02	0.10	-0.21	0.832	
Triglyceride levels	0.17	0.05	3.28	0.002	
**Time begging instead of "Treatment" model**					
Intercept	1.92	0.09	20.2	<0.001	
Time begging	-0.14	0.06	-2.27	0.036	0.182
Initial MDA (residuals)	0.42	0.10	4.37	<0.001	
Initial mass	-0.01	0.10	-0.14	0.893	
Final triglyceride levels	0.33	0.10	3.41	0.002	
**Within-Dyad differences model**					
Intercept	-0.24	0.13	-1.85	0.096	
Dif. time begging	-0.16	0.12	-1.34	0.213	0.525
Dif. initial MDA (residuals)	0.13	0.15	0.85	0.415	
Dif. initial mass	-0.19	0.12	-1.55	0.146	
Dif. final triglyceride levels	0.35	0.16	2.25	0.042	

TABLE S6: Estimates (± SE), *t* values and *P*-values for models explaining differences in MDA levels with and without correcting by triglyceride levels after incorporating mass gain and PHA as covariates. "*Treatment*" and "*Time*" are categorical factors with "*LB*" and "*initial levels*" as reference groups.

	**Estimate**	**SE**	**t**	***P***
**MDA model**				
Intercept	1.75	0.17	10.5	<0.001
Treatment × Time	-0.12	0.16	-0.79	0.435
Treatment	-0.10	0.11	-0.89	0.378
Time	0.51	0.11	4.62	<0.001
Mass gain	0.11	0.09	1.14	0.263
PHA	<0.01	0.07	0.01	0.992
**MDA model adding triglyceride levels as covariate**				
Intercept	1.69	0.16	10.4	<0.001
Treatment × Time	-0.18	0.16	-1.17	0.249
Treatment	-0.05	0.11	-0.42	0.674
Time	0.36	0.11	3.20	0.003
Mass gain	-0.11	0.11	-0.96	0.343
PHA	0.09	0.08	1.14	0.266
Triglyceride levels	0.17	0.05	3.35	0.002

TABLE S7: Estimates (± SE), *t*-values or (z values for total WBC and lysis analyses) and *P*-values for models explaining final values of body mass, CORT levels, immunological and oxidative stress markers as a function of time spent begging, initial values of the measured variable (except for PHA, that was only measured at the end of the experiment) and initial body mass. We also provide corrected *P*-values (Benjamini-Hochberg method) for the effect of the average nestling time begging on each model. Initial levels of TEAC were computed as residuals of TEAC corrected by uric acid levels.

	Estimate	SE	t/z	*P*	Corrected *P*
**Growth**					
**Final mass**					
Intercept	64.5	0.76	84.6	<0.001	
Time begging	<0.01	0.28	0.01	0.989	0.989
Initial mass	5.42	0.59	9.13	<0.001	
**Body mass day 9**					
Intercept	57.7	0.37	155	<0.001	
Time begging	-0.03	0.16	-0.20	0.843	0.989
Initial mass day 9	5.91	0.30	19.9	<0.001	
**Body mass day 10**					
Intercept	64.1	0.18	351	<0.001	
Time begging	0.21	0.18	1.18	0.249	0.746
Initial mass day 10	6.48	0.19	34.6	<0.001	
**CORT**					
Intercept	0.19	0.02	9.62	<0.001	
Time begging	-0.02	0.01	-1.35	0.189	0.189
Initial CORT	-0.02	0.02	-1.18	0.244	
Initial mass	-0.04	0.02	-2.13	0.041	
Handling time	-0.01	0.01	-0.54	0.590	
**Immunity**					
**PHA skin test**					
Intercept	1.51	0.06	26.3	<0.001	
Time begging	-0.08	0.04	-2.23	0.034	0.236
Initial mass	0.02	0.06	0.39	0.701	
**Total WBC count**					
Intercept	24.1	2.63	9.17	<0.001	
Time begging	-1.42	1.83	-0.77	0.445	0.642
Initial Total WBC	1.27	2.32	0.55	0.586	
Initial mass	-0.83	2.51	-0.33	0.742	

**H:L ratio**					
Intercept	0.56	0.03	17.7	<0.001	
Time begging	-0.03	0.03	-1.26	0.220	0.568
Initial H:L ratio	-0.01	0.03	-0.43	0.668	
Initial mass	-0.07	0.03	-2.38	0.024	
**Lysis (prob)**					
Intercept	-1.78	0.78	-2.27	0.023	
Time begging	0.30	0.40	0.74	0.459	0.642
Initial lysis (prob)	1.93	0.99	1.94	0.052	
Initial mass	0.33	0.49	0.66	0.508	
**Agglutination**					
Intercept	0.85	0.06	14.3	<0.001	
Time begging	-0.02	0.04	-0.41	0.687	0.687
Initial agluttination	0.04	0.05	0.88	0.384	
Initial mass	0.10	0.05	1.99	0.057	
**Haptoglobin**					
Intercept	0.13	0.02	8.71	<0.001	
Time begging	<0.01	0.01	0.49	0.625	0.687
Initial haptoglobin	0.03	0.01	2.92	0.005	
Initial mass	<0.01	0.01	0.34	0.734	
**Bacterial killing capacity**					
Intercept	-1.63	0.18	-9.23	<0.001	
Time begging	0.21	0.17	1.22	0.244	0.568
Initial Dead cells	1.15	0.17	6.78	<0.001	
Initial mass	0.31	0.19	1.67	0.116	
**Oxidative stress**					
**MDA**					
Intercept	2.14	0.12	17.3	<0.001	
Time begging	-0.09	0.06	-1.42	0.169	0.519
Initial MDA	0.44	0.11	3.94	<0.001	
Initial mass	0.05	0.09	0.57	0.577	
**TEAC**					
Intercept	0.77	0.04	20.2	<0.001	
Time begging	0.02	0.02	0.66	0.519	0.519
Initial TEAC (residuals)	-0.02	0.03	-0.69	0.508	
Initial mass	<-0.01	0.03	-0.01	0.990	
Final uric acid levels	0.13	0.03	4.78	<0.001	
**OXY**					
Intercept	5.89	0.01	782	<0.001	
Time begging	<0.01	<0.01	0.89	0.388	0.519
Initial OXY	<-0.01	<0.01	-0.21	0.836	
Initial mass	<-0.01	0.01	-0.31	0.762	

**tGSH**					
Intercept	2.70	0.06	43.2	<0.001	
Time begging	-0.05	0.04	-1.12	0.274	0.519
Initial tGSH	0.33	0.05	6.09	<0.001	
Initial mass	0.13	0.05	2.40	0.026	
**GSH:GSSG ratio**					
Intercept	7.89	0.58	13.6	<0.001	
Time begging	-0.42	0.56	-0.74	0.466	0.519
Initial GSH:GSSG ratio	0.25	0.60	0.42	0.678	
Initial mass	-0.24	0.58	-0.41	0.684	

TABLE S8: Estimates (± SE), *t* values and *P*-values for models explaining HB-LB differences within-dyads in body mass, CORT levels, immunological and oxidative stress markers as a function of HB-LB differences in time spent begging and initial mass. We also provide corrected *P*-values (Benjamini-Hochberg method) for the effect of the difference in time spent begging. Initial levels of TEAC were computed as residuals of TEAC corrected by uric acid levels.

	Estimate	SE	t	*P*	Corrected *P*
**Growth**					
**Dif. final mass**					
Intercept	-0.20	0.63	-0.32	0.755	
Dif. time begging	0.31	0.61	0.51	0.616	0.920
Dif. Initial mass	2.96	0.58	5.15	<0.001	
**Dif. body mass day 9**					
Intercept	-0.14	0.35	-0.39	0.701	
Dif. time begging	-0.03	0.34	-0.10	0.920	0.920
Dif. Initial mass day 9	3.41	0.33	10.4	<0.001	
**Dif. body mass day 10**					
Intercept	-0.02	0.41	-0.04	0.967	
Dif. time begging	0.10	0.42	0.23	0.819	0.920
Dif. Initial mass day 10	3.50	0.43	8.15	<0.001	
**Dif. CORT**					
Intercept	-0.09	0.11	-0.81	0.434	
Dif. time begging	0.02	0.12	0.20	0.845	0.845
Dif. initial CORT	<-0.01	0.12	-0.03	0.979	
Dif. initial mass	-0.12	0.11	-1.05	0.305	
Dif. handling time	0.09	0.12	0.75	0.463	
**Immunity**					
**Dif. PHA skin test**					
Intercept	-0.12	0.07	-1.73	0.109	
Dif. time begging	-0.15	0.07	-2.15	0.042	0.190
Dif. initial mass	-0.10	0.07	-1.52	0.143	
**Dif. total WBC count**					
Intercept	-1.67	3.53	-0.47	0.646	
Dif. time begging	-7.28	3.65	-1.99	0.058	0.190
Dif. initial Total WBC	-1.76	3.76	-0.47	0.645	
Dif. initial mass	-2.23	3.61	-0.62	0.543	
**Dif. H:L ratio**					
Intercept	-0.15	0.24	-0.63	0.545	
Dif. time begging	0.07	0.25	0.30	0.770	0.825

Dif. initial H:L ratio	-0.07	0.26	0.27	0.792	
Dif. initial mass	-0.26	0.24	-1.09	0.289	
**Dif. lysis (prob)**					
Intercept	0.12	0.13	0.95	0.361	
Dif. time begging	-0.23	0.13	-1.83	0.082	0.190
Dif. initial lysis (prob)	0.08	0.11	0.80	0.434	
Dif. initial mass	0.03	0.10	0.32	0.754	
**Dif. agglutination**					
Intercept	<0.01	0.26	<0.01	0.998	
Dif. time begging	-0.29	0.18	-1.67	0.120	0.210
Dif. initial agluttination	0.22	0.15	1.51	0.157	
Dif. initial mass	-0.07	0.13	-0.52	0.613	
**Dif. haptoglobin**					
Intercept	0.01	0.02	0.79	0.447	
Dif. time begging	-0.01	0.02	-0.45	0.659	0.825
Dif. initial haptoglobin	0.04	0.02	2.44	0.023	
Dif. initial mass	<-0.01	0.02	-0.19	0.855	
**Dif. bacterial killing capacity**					
Intercept	5.22	7.14	0.73	0.483	
Dif. time begging	-2.70	11.9	-0.23	0.825	0.825
Dif. initial Dead cells	-0.03	6.50	-0.01	0.996	
Dif. initial mass	3.02	5.25	0.58	0.580	
**Oxidative stress**					
**Dif. MDA**					
Intercept	-0.23	0.13	-1.74	0.108	
Dif. time begging	-0.09	0.13	-0.69	0.497	0.672
Dif. initial MDA	<0.01	0.13	0.01	0.992	
Dif. initial mass	-0.07	0.12	-0.58	0.569	
**Dif. TEAC**					
Intercept	0.07	0.06	1.13	0.290	
Dif. time begging	-0.07	0.07	-1.06	0.315	0.672
Dif. initial TEAC (residuals)	0.01	0.05	0.20	0.851	
Dif. initial mass	<-0.01	0.04	-0.01	0.994	
Dif. final uric acid levels	0.17	0.05	3.35	0.008	
**Dif. OXY**					
Intercept	29.6	9.27	3.19	0.011	
Dif. time begging	-1.17	0.37	-3.15	0.014	0.070
Dif. initial OXY	-0.22	1.41	-0.15	0.883	
Dif. initial mass	-3.88	1.48	-2.62	0.028	

**Dif. tGSH**					
Intercept	-0.13	0.11	-1.25	0.240	
Dif. time begging	-0.04	0.09	-0.43	0.672	0.672
Dif. initial tGSH	0.23	0.09	2.55	0.022	
Dif. initial mass	0.10	0.08	1.37	0.193	
**Dif. GSH:GSSG ratio**					
Intercept	-1.19	1.19	-1.00	0.343	
Dif. time begging	-0.54	1.11	-0.48	0.635	0.672
Dif. initial GSH:GSSG ratio	-1.47	1.05	-1.40	0.184	
Dif. initial mass	1.40	0.90	1.55	0.146	

TABLE S9: Mean ± SE of final values of body mass, CORT levels, immunological and oxidative stress markers measured in this study for low-begging (LB) and high-begging (HB) experimental groups. We also provide effect size computed as Hedges's g for dependent samples with 95% confidence intervals (CI) for mean differences between LB and HB nestlings. For lysis capacity, we provide odds ratio (OR) instead of Hedges's g. TEAC was computed as residuals of TEAC corrected by uric acid levels.

	LB	HB	Hedges's g/OR	95% CI
**Growth**				
Body mass day 9 (g)	57.6 ± 1.17	57.5 ± 1.15	-0.03	-0.55, 0.48
Body mass day 10 (g)	64.1 ± 1.21	64.1 ± 1.28	<-0.01	-0.52, 0.51
Total body mass gain	15.5 ± 0.75	15.4 ± 0.69	-0.01	-0.53, 0.50
**CORT (ng/ml)**	0.44 ± 0.08	0.35 ± 0.08	-0.17	-0.69, 0.36
**Immunological assays**				
PHA skin test (mm)	1.57 ± 0.07	1.45 ± 0.06	-0.30	-0.82, 0.22
Total WBC count (cells/10,000 erythrocytes)	24.8 ± 3.03	23.1 ± 2.81	-0.09	-0.60, 0.43
H:L ratio	1.28 ± 0.15	1.13 ± 0.16	-0.12	-0.64, 0.39
Lysis (prob)	0.27 ± 0.09	0.38 ± 0.10	1.70	0.53, 5.48
Agglutination (titers)	1.50 ± 0.15	1.50 ± 0.17	0.00	-0.54, 0.54
Haptoglobin (mg/ml)	0.18 ± 0.03	0.19 ± 0.03	0.14	-0.38, 0.65
Bacterial killing capacity (% *E. coli* dead)	21.5 ± 5.39	26.5 ± 6.17	0.28	-0.44, 1.00
**Oxidative stress**				
MDA (µmol/L)	2.25 ± 0.17	2.02± 0.15	-0.40	-0.97, 0.17
TEAC (residuals) (mmol Trolox Eq/L)	-0.07 ± 0.03	-0.05 ± 0.04	0.11	-0.54, 0.76
OXY (µmol of HClO neutralized)	361 ± 2.69	364 ± 2.60	0.31	-0.33, 0.95
tGSH (mmol/g)	2.73 ± 0.11	2.66 ± 0.09	-0.14	-0.74, 0.47
GSH:GSSG ratio	8.35 ± 0.72	7.45 ± 0.75	-0.19	-0.82, 0.43
